# Luteolin-Rich Extract from *Harrisonia perforata* (Blanco) Merr. Root Alleviates SARS-CoV-2 Spike Protein-Stimulated Lung Inflammation via Inhibition of MAPK/NLRP3 Inflammasome Signaling Pathways

**DOI:** 10.3390/life15071077

**Published:** 2025-07-05

**Authors:** Warathit Semmarath, Punnida Arjsri, Kamonwan Srisawad, Sonthaya Umsumarng, Pornngarm Dejkriengkraikul

**Affiliations:** 1 Akkhraratchakumari Veterinary College, Walailak University, Nakhon Si Thammarat 80160, Thailand; warathit.se@wu.ac.th; 2 Centre for One Health, Walailak University, Nakhon Si Thammarat 80160, Thailand; 3 Department of Biochemistry, Faculty of Medicine, Chiang Mai University, Chiang Mai 50200, Thailand; punnida.dream@gmail.com (P.A.); kamonwan.sri@cmu.ac.th (K.S.); 4 Anticarcinogenesis and Apoptosis Research Cluster, Faculty of Medicine, Chiang Mai University, Chiang Mai 50200, Thailand; 5 Faculty of Veterinary Medicine, Chiang Mai University, Chiang Mai 50100, Thailand; sonthaya.u@cmu.ac.th

**Keywords:** *Harrisonia perforata*, luteolin, spike protein, Wuhan-origin: Omicron variant, NLRP3 inflammasome, MAPK pathway

## Abstract

The COVID-19-related long-standing effect or Post-Acute Sequelae of COVID-19 (PASC) is often associated with NLRP3 inflammasome activation in pulmonary inflammation elicited by SARS-CoV-2 spike proteins. Spike proteins engage toll-like receptors (TLRs) in respiratory epithelial cells, leading to excessive cytokine production. Given the need for effective therapeutic strategies to mitigate spike protein-stimulated lung inflammation, we examined the anti-inflammatory properties of luteolin and ethanolic extract from *Harrisonia perforata* (Blanco) Merr. root. The ethanolic extract of *H. perforata* root (HPEE) contained a high concentration of luteolin flavonoid (143.53 ± 1.58 mg/g extract). Both HPEE (25–100 μg/mL) and luteolin (4.5–36 μM) significantly inhibited inflammation stimulated by the Wuhan (W) and Omicron (O) spike protein S1, as evidenced by a dose-dependent significant decrease in IL-6, IL-1β, and IL-18 secretion in A549 lung epithelial cells (*p* < 0.05). Furthermore, pretreatment with HPEE or luteolin prior to spike protein exposure (100 ng/mL) significantly, in a dose-dependent manner, repressed the inflammatory mRNA expression (*p* < 0.05). Mechanistic study revealed that HPEE and luteolin suppressed NLRP3 inflammasome signaling activation by reducing their machinery protein expressions. Additionally, they inhibited the ERK/JNK/p38 MAPK signaling activation, resulting in decreased inflammatory mRNA expression and cytokine release. These findings suggest that *H. perforata* root extract and its major flavonoid luteolin exert potent anti-inflammatory effects and may offer therapeutic potential against spike protein-induced lung inflammation.

## 1. Introduction

The COVID-19 global pandemic was caused by severe acute respiratory syndrome coronavirus 2, or SARS-CoV-2. While vaccinations have effectively reduced disease severity and transmission, many COVID-19 patients continue to experience a wide range of pathological symptoms far from the acute infectious stage, including fever, respiratory distress, gastrointestinal issues, and neurological complications [1,2]. The pandemic highlighted the need to advance personalized and precision medicine by considering individual differences, environmental factors, and social determinants of health [3,4]. It quickly became evident that individuals over 65 years were at greater risk for COVID-19 disease outcome severity, alongside obesity, type 2 diabetes, and other non-communicable chronic diseases [5,6]. These findings contributed to the development of targeted therapies for high-risk populations and stimulated ongoing research into alternative therapeutic strategies [7,8].

SARS-CoV-2 primarily targets the lower respiratory system or pulmonary tissue, with infection facilitated by binding of its spike glycoprotein to receptors on airway epithelial cells. These cells express two critical receptors, Angiotensin-converting enzyme 2 (ACE-2) and Transmembrane serine protease 2, which facilitate attachment and viral entry into the human host cells [9,10]. Furthermore, the SARS-CoV-2 spike protein shares similar antigenic determinants (epitopes) with humans, leading to auto-immune reactivity along with the stimulation of toll-like receptors (TLRs) [11]. This interaction generates an innate immune cascade, resulting in the instigation of numerous inflammatory signaling pathways, including the NLRP3 inflammasome, and pro-inflammatory cytokine releases [12].

Nowadays, genetic mutations in the spike protein part of SARS-CoV-2 have enhanced its ability to recognize human receptors, leading to the occurrence of genetically distinct variants, comprising of α: Alpha (B.1.1.7), β: Beta (B.1.351), γ: Gamma (P.1), δ: Delta (B.1.617), and ο: Omicron (B.1.1.529) [13,14,15]. The mutations not only improve viral binding efficiency but also contribute to immune evasion. Additionally, remaining antigens of the spike protein can persist in the circulatory system and upper or lower respiratory tissues, sustaining a sub-chronic inflammatory state and potentially contributing to long-term chronic inflammatory lung disorders [16,17]. Given these encounters, advances in therapeutic or preventative approaches targeting spike protein-stimulated inflammation are crucial for managing long COVID-19 symptoms.

Controlling spike protein-mediated inflammation is essential for preventing chronic lung inflammation in long COVID-19 patients. Recently, naturally occurring phytochemicals have gained attention for their potential therapeutic effects against COVID-19-associated inflammation [18,19]. Bioactive compounds such as luteolin from *Perilla frutescens* [20] have demonstrated anti-inflammation properties by inhibiting spike protein-stimulated inflammatory responses in A549 lung and THP-1 macrophage models. *Harrisonia perforata* (Blanco) Merr. (*H. perforata*), family Rutaceae, is a commonly cultivated plant in Indonesia, Malaysia, as well as Thailand [21,22]. In Thai traditional alternative medicine, *H. perforata* is a key component of Benjalokawichien or Ha-Rak Thai remedy, commonly used for its anti-pyretic and anti-allergic properties. The dried root of *H. perforata* has long been used to treat fever and inflammation [23]. Previously, ethanolic extract from the root of *H. perforata* displayed anti-allergic activity by inhibiting RBL-2H3 cells’ β-hexosaminidase release and possessed anti-inflammation properties by suppressing nitric oxide production in RAW264.7 macrophages [24,25]. Therefore, the aforementioned findings suggested *H. perforata* as a potential inhibitor of spike protein-stimulated lung inflammation in long COVID-19 patients.

The objectives of this study were to explore the inhibitory properties of *H. perforata* root extract and its bioactive compound, luteolin, on spike protein-stimulated inflammation in lung epithelial cells. Specifically, it evaluates their impact on inflammatory responses in A549 lung epithelial cells following exposure to spike proteins S1 from the Wuhan and Omicron variants. Additionally, this study explored the underlying mechanisms of their anti-inflammation activity, focusing on MAPK and NLRP3 inflammasome signaling regulations.

## 2. Materials and Methods

### 2.1. Chemicals and Reagents

Catechin, epicatechin, hyperoside, rutin, quercitrin, quercetin, luteolin, and apigenin were provided by MedChemExpress Company (Monmouth Junction, NJ, USA). Recombinant human coronavirus SARS-CoV-2 spike glycoprotein S1 (Active) (Wuhan-original, Cat. No. ab273068) and Recombinant human coronavirus SARS-CoV-2 spike glycoprotein S1 (B.1.1.529/Omicron, Cat. No. ab290828) were provided by Abcam (Cambridge, UK). Dulbecco’s Modified Eagle Medium (DMEM), trypsin enzyme concentrated 10x, penicillin–streptomycin, and fetal bovine serum (FBS) were provided by Gibco BRL (Grand Island, NY, USA). The QIAzol lysis reagent was provided by Qiagen (Valencia, CA, USA). qPCR Master Mix, ReverTra Ace^®^, was provided by Toyobo Co., Ltd. (Osaka, Japan). SensiFAST^TM^ SYBR^®^ Lo-ROX Kit was provided by Meridian Bioscience^®^ (Cincinnati, OH, USA). IL-6, IL-1β, and IL-18 Enzyme-Linked Immunosorbent Assay (ELISA) test kits were provided by BioLegend (San Diego, CA, USA). The primary antibodies of NLRP3, ASC, caspase-1, p-ERK1/2, ERK1/2, p-JNK, JNK, p-p38, and p38, along with horseradish peroxidase (HRP)-conjugated goat anti-mouse and rabbit IgG, were provided by Cell Signaling Technology (Danvers, MA, USA). The sulforhodamine B (SRB) and primary antibody of β-actin were provided by Sigma-Aldrich (St. Louis, MO, USA).

### 2.2. Herb Materials and Extraction Technique

*Harrisonia perforata* (Blanco) Merr. was cultivated and harvested in June 2022 in a home-grown farm of Phitsanulok, Thailand. The voucher specimen (certified No. RT046) of *H. perforata* was issued by the herbarium at the Flora of Thailand, Faculty of Pharmacy, Chiang Mai University, Thailand.

Regarding the ethanolic extraction, initially, 500 g of *H. perforata* dried roots were soaked in 6 L of ethanol (80% *v*/*v*) and were stirred for 48 h. The solvent was filtered and concentrated with rotary vacuum evaporator at a temperature of 56 °C to achieve the ethanolic extract. The extract was re-suspended in DI water and underwent the process of freeze-drying to obtain the *H. perforata* ethanolic extracts (HPEEs). HPEEs were kept at −20 °C.

### 2.3. Phytochemical Compounds Determination of HPEE

The flavonoid amount was assessed using the aluminum chloride (AlCl_3_) colorimetric assay [26]. The absorbance obtained from UV–visible spectrophotometry was set at 510 nm. The milligram (mg) catechin equivalents per gram of extract (mg catechin/g extract) were used to represent the total flavonoid content unit.

The High-Performance Liquid Chromatography (HPLC) was performed to identify the flavonoid compounds in HPEE using these flavonoid standards: catechin, epicatechin, hyperoside, rutin, quercitrin, quercetin, luteolin, and apigenin. The HPLC conditions were modified from a previously described protocol [27]. The 0.2% formic acid in water (A) and 100% methanol (B) were used as mobile phases under gradient conditions as described in Table 1.

The samples were analyzed using an Agilent Infinity 1260 HPLC system (Santa Clara, CA, USA) with a Zorbax Eclipse Plus C18 column (250 mm × 4.6 mm, 5 µm). The detection UV absorbance was fixed at 270 nm wavelength, with a flow rate of 1.0 mL/min at 40 °C. HPEE concentration of 10 mg/mL and flavonoid standard concentrations of 0–0.1 mg/mL were used with the injection volumes of 10 μL.

### 2.4. Cell Cultures

The A549 human lung epithelial cells were purchased from ATCC. Primary dermal fibroblasts from human origin were obtained from an abdominal scar tissue of a cesarean section operated at the surgery department of Chiang Mai Maharaj Hospital, Chiang Mai, Thailand. The dermal fibroblast cells (DFCs) were isolated using a previously described protocol [28]. Briefly, primary human skin fibroblasts were aseptically isolated from an abdominal scar of the skin, adipose tissue was removed, and the sample was soaked in DMEM containing penicillin and streptomycin. The tissue was then cultured in DMEM with 1% protease at 4 °C for 48 h. After removal of the epidermal layer, fibroblasts were isolated from the dermis and cultured in 10% FBS of DMEM with 2 mM L-glutamine, 50 U/mL penicillin, and 50 μg/mL streptomycin. Both cells were maintained as a monolayer in DMEM supplemented with 10% FBS, L-glutamine, and aforementioned antibiotics in a 5% CO_2_ incubator at 37 °C. The cells were collected when confluency percentage reached 70% but not more than 80%.

### 2.5. Cytotoxicity Assessment of HPEE and Luteolin on A549 Cells and Primary Fibroblasts

The effects of HPEE and luteolin on A549 lung and DFCs cells’ viability were assessed using the sulforhodamine B (SRB) colorimetric assay. A549 cells were seeded with the culture medium (3 × 10^3^ cells per well) and incubated with HPEE (0 to 200 μg/mL) or commercial luteolin (0 to 140 μM) solubilized in DMSO for 24 and 48 h. After that, 10% (*w*/*v*) trichloroacetic acid was added to each well and incubated for 1 h at 4 °C. Then, SRB dye at the concentration of 0.054% *w*/*v* was added to the well and was incubated for additional 30 min at 25 °C. Then, SRB dye was removed and rinsed with 1% *v*/*v* acetic acid 3 times. Then, SRB dye was dissolved in Tris-base solution (10 mM). The cells were subjected to absorbance measurement at 510 nm wavelength. A percentage relative to the control was calculated to represent the cell viability.

### 2.6. Effects of HPEE and Luteolin on Cytokine Secretions in A549 Cells

The A549 culture medium samples were used to detect levels of IL-6, IL-1β, and IL-18 using an Enzyme-Linked Immunosorbent Assay (ELISA) kit following the manufacturer’s protocol. A549 cells were seeded in a 6-well culture plate (3 × 10^5^ cells/well) and pretreated with HPEE (0–100 μg/mL) or luteolin (0–36 μM) solubilized in DMSO for 24 h. After that, cells were stimulated to either Wuhan-original (W) or Omicron (O) variant of spike proteins S1 (100 ng/mL) for the duration of 6 h. The culture medium was collected for ELISA kit cytokine secretions using the absorbance at 450 and 570 nm. IL-6, IL-1β, and IL-18 cytokine secretion levels were performed in triplicate and calculated with the corresponding standard calibration curves.

### 2.7. Effects of HPEE and Luteolin on Inflammatory Gene Expressions

To study the properties of HPEE and luteolin on inflammatory gene expressions, A549 cells were seeded and treated under the same conditions as Section 2.6. Total RNA was extracted using Qiazol reagent and equipped for reverse transcription using a Mastercycler^®^ nexus gradient machine. The quantitative real-time PCR was analyzed using the qRT-PCR ABITM 7500 Fast & 7500 Real-Time PCR machine (Thermo Fisher Scientific, Waltham, MA, USA). mRNA expression levels were determined using the QuantStudio 6 Flex real-time PCR software v1.0 (Applied Biosystems, Waltham, MA, USA) and were calculated using the 2^−ΔΔCT^ method with normalization of GAPDH housekeeping gene and control samples. The primer sequences used in this study for IL-6 (Bio Basic Canada Inc., Markham, ON, Canada), NLRP3, IL-1β, IL-18, and GAPDH (Humanizing Genomics Macrogen, Ge-umcheongu, Seoul, Republic of Korea) are shown in the Supplementary Materials (Table S1) [20].

### 2.8. Effects of HPEE and Luteolin on Spike Protein-Dependent-MAPK/NLRP3 Inflammasome Activation in A549 Epithelial Cells

The effect of HPEE and luteolin on MAPK pathway and NLRP3 inflammasome machinery proteins in Spike S1-stimulated airway epithelial cells inflammation was determined using Western blotting analysis to determine the protein expressions. A549 cells were seeded and treated under the same conditions as Section 2.6 and Section 2.7. After treatment, cells were harvested and lysed with RIPA buffer, and protein concentration was determined using the Bradford assay. Whole-cell lysates were subjected to 12% SDS-PAGE for protein separation and subsequently transferred to nitrocellulose membranes. The membranes were then incubated at 4 °C for 12 h with primary antibodies of NLRP3 (1:1000), ASC (1:2000), caspase-1 (1:1000), p-ERK1/2 (1:1000), ERK1/2 (1:2000), p-JNK (1:1000), JNK (1:2000), p-p38 (1:1000), or p38 (1:2000). Bound antibodies were detected using a chemiluminescent detection system and iBright™ CL-1500 imaging system. Band intensity was analyzed using ImageJ software version 1.410.

### 2.9. Statistical Analysis

All data in each experiment was carried out at least three triplicate. Data was displayed as mean ($\bar{X}$) ± standard deviation (S.D.) values. The independent t-test was used for statistical testing. The *p*-values of 0.05, 0.01, and 0.001 were considered statistically significant. Statistical analysis was performed using Prism software version 8.0.

## 3. Results

### 3.1. Extraction and Phytochemical Characteristics of HPEE

To study the anti-inflammation activities of the flavonoid-containing plant upon SARS-CoV-2 spike protein induction, the ethanolic extract of *H. perforata* (HPEE) was first prepared. The percentage yield of HPEE was recorded at 6.05 ± 0.98% (*w*/*w*). HPEE was then analyzed for its active flavonoid compounds using total flavonoid content screening by the AlCl_3_ colorimetric assay and HPLC technique.

**Figure 1:**
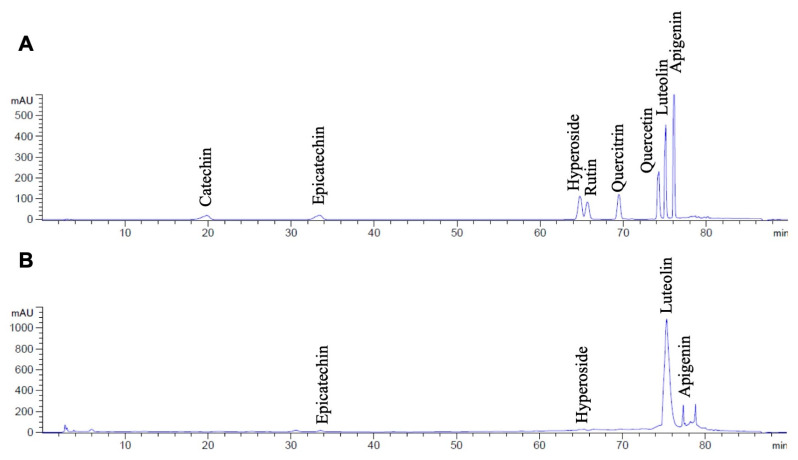
HPLC chromatogram of the flavonoid compounds (catechin, epicatechin, hyperoside, rutin, quercitrin, quercetin, luteolin, and apigenin) according to their retention time (100 μg/mL for each standard: (**A**). HPLC chromatogram of HPEE at (10,000 μg/mL) (**B**). The HPLC chromatograms were evaluated using reversed-phase C18 column in gradient conditions. Detection wavelength was 270 nm.

Regarding the phytochemical characteristics of HPEE, as shown in Table 2, HPEE contained high amounts of total flavonoid compounds (152.17 ± 14.56 mg of catechin equivalents/g extract). The key flavonoid compounds, including catechin, epicatechin, hyperoside, rutin, quercitrin, quercetin, luteolin, and apigenin, were identified and quantified in HPEE using HPLC analysis, as shown in the HPLC chromatograms for standard compounds and HPEE in Figure 1. Luteolin was identified as the major compound in HPEE, with a concentration of 143.53 ± 1.58 mg/g extract. In contrast, epicatechin (8.22 ± 0.57 mg/g extract), hyperoside (3.33 ± 0.70 mg/g extract), and apigenin (2.34 ± 0.22 mg/g extract) were present in significantly lower amounts. These results indicate that luteolin is the predominant flavonoid compound in HPEE. Therefore, HPEE, together with luteolin, was further used in the subsequent experiments for the inhibitory effects against Spike proteins S1-stimulated lung cells inflammation.

### 3.2. Cell Viability Effects of HPEE on A549 Lung Epithelial Cells and Primary Dermal Fibroblasts

**Figure 2:**
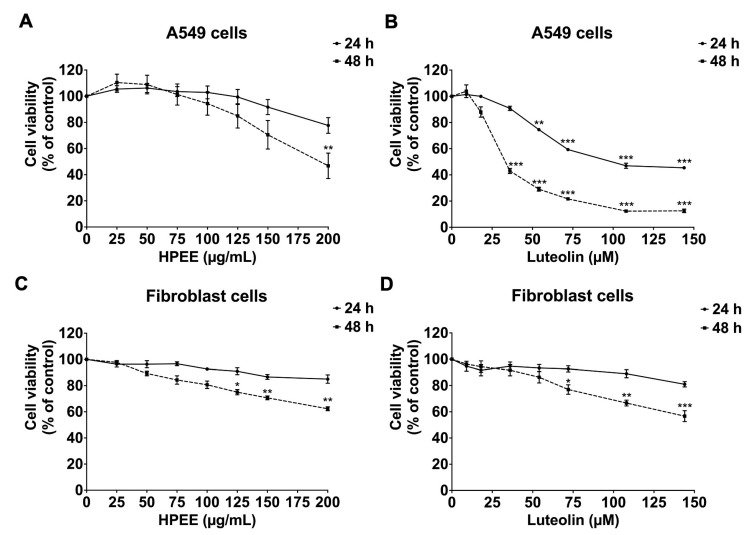
Cytotoxicity assessment of HPEE and luteolin on A549 lung cells (**A**,**B**) and dermal human fibroblasts (**C**,**D**). A549 cells and fibroblast cells (3 × 10^3^ cells/well) were treated with increasing concentrations of HPEE (0 to 200 µg/mL) and luteolin (0 to 140 µM) for 24 h and 48 h. Cell viability was assessed using the SRB assay. Data are presented as mean ± S.D. from at least three independent experiments. * *p* < 0.05, ** *p* < 0.01 and *** *p* < 0.001 = statistical significance compared to the control for the respective incubation time.

Before assessing the anti-inflammation properties of HPEE and luteolin, their cytotoxic effects on A549 lung epithelial were assessed using the SRB assay. Cell viability was determined following incubation with HPEE (0–200 μg/mL) and luteolin (0–140 µM) for 24 and 48 h. The resulting data is presented in Figure 2A,B, with IC_50_ values summarized in Table 3.

The findings indicated that HPEE did not exhibit significant cytotoxicity within the tested concentration range at either 24 h or 48 h, except at 200 μg/mL, where a notable reduction in cell viability was observed at 48 h. In contrast, luteolin significantly reduced A549 cell viability at concentrations exceeding 50 μM, with viability dropping below 80% after 24 h of treatment. The IC_50_ values for HPEE on A549 cells were >200 μg/mL at 24 h and 178 ± 26.87 μg/mL at 48 h. Meanwhile, luteolin exhibited higher cytotoxicity, with IC_50_ values of 101 ± 7.57 μM at 24 h and 34 ± 1.53 μM at 48 h. Given that flavonoids are naturally occurring compounds with minimal side effects [20,29], we also assessed the cytotoxicity of HPEE and luteolin on normal primary human dermal fibroblasts. As shown in Figure 2C,D, neither HPEE nor Luteolin exhibited cytotoxicity in fibroblasts at 24 or 48 h, with IC_50_ values exceeding 200 μg/mL (HPEE) and 140 µM (luteolin).

Based on these cell viability results, non-toxic concentrations (with cell viability more than 80%) of HPEE (0–100 μg/mL) and luteolin (0–36 µM) were selected for evaluating their anti-inflammation effects on A549 cells, specifically in the context of spike protein-stimulated NLRP3-inflammasome activation.

### 3.3. Effects of HPEE and Luteolin on Proinflammatory Cytokine Releases in SARS-CoV-2 Spike Proteins S1-Stimulated A549 Lung Cell Inflammation

To establish the inflammatory trigger conditions in A549 cells, we first determined the optimum required time for an inflammatory response regarding spike protein S1 induction for both the Wuhan-original (W) and Omicron (O) variants. Based on previous studies, a spike protein S1 concentration of 100 ng/mL was selected as a reference for inducing inflammatory responses in lung epithelial cells [30,31]. As shown in Figures S1 and S2, our laboratory findings confirmed that the optimum time for response was determined by measuring the increased expression of all observed inflammatory mRNAs and cytokine releases in A549 cells following spike protein S1 stimulation. These inflammatory markers were consistently upregulated in response to both W and O spike proteins, highlighting their role in SARS-CoV-2-mediated lung inflammation [32,33].

**Figure 3:**
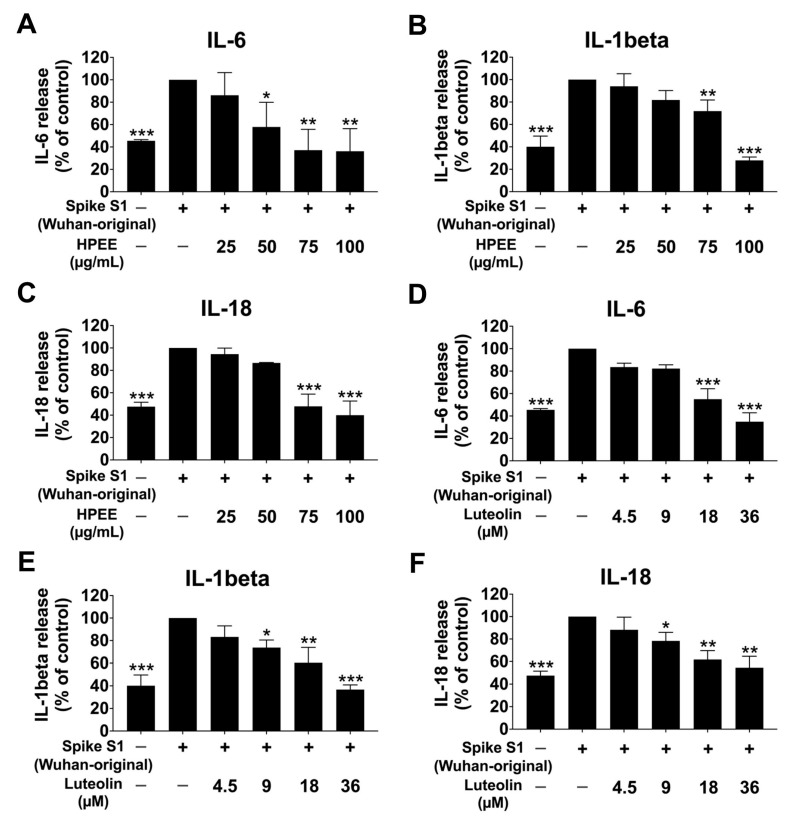
Effects of HPEE and luteolin on cytokine production in SARS-CoV-2 Spike S1 (Wuhan-original)-stimulated A549 cells. Cells were pretreated with HPEE at a concentration of 25–100 μg/mL (**A**–**C**) and luteolin at concentrations of 4.5–36 μM (**D**–**F**) for 24 h. Then, the cells were exposed to 100 ng/mL of SARS-CoV-2 Spike for 6 h. The data (mean ± S.D.) was obtained from at least three independent experiments. * *p* < 0.05, ** *p* < 0.01, and *** *p* < 0.001 = statistical significance compared with the Spike S1-stimulated control group.

**Figure 4:**
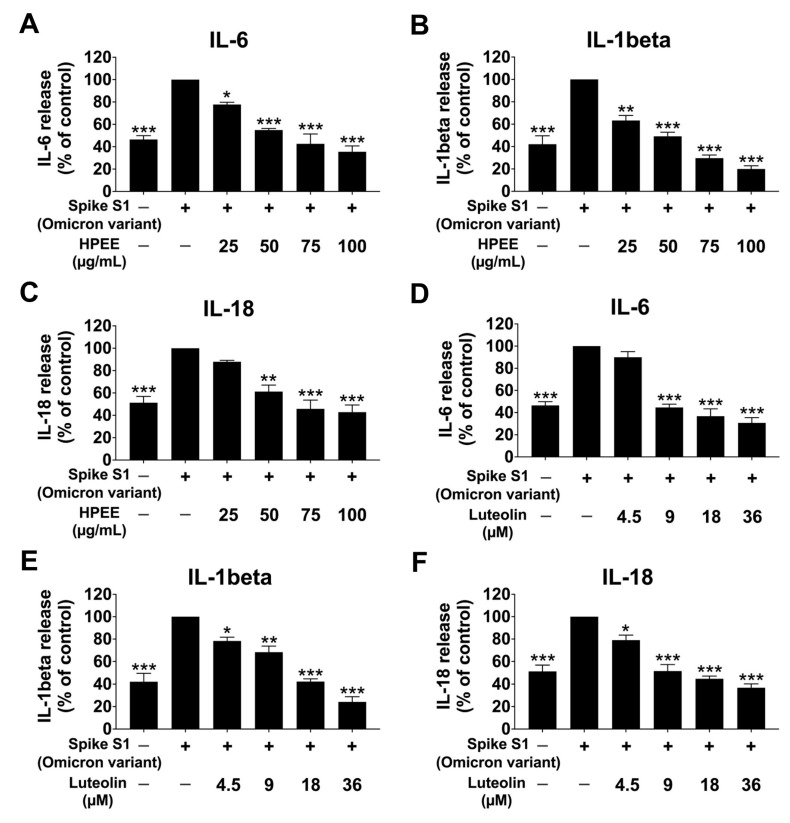
Effects of HPEE and luteolin on cytokine production in SARS-CoV-2 Spike S1 (Omicron variants)-stimulated A549 cells. Cells were pretreated with HPEE at a concentration of 25–100 μg/mL (**A**–**C**) and luteolin at concentrations of 4.5–36 μM (**D**–**F**) for 24 h. Then, the cells were exposed to 100 ng/mL of SARS-CoV-2 Spike for 6 h. The data (mean ± S.D.) was obtained from at least three independent experiments. * *p* < 0.05, ** *p* < 0.01, and *** *p* < 0.001 = statistical significance compared with the Spike S1-stimulated control group.

Regarding the anti-inflammation effects of HPEE and luteolin, we examined their ability to suppress cytokine release in spike protein S1-stimulated A549 cells. Following stimulation with spike protein S1 from both W (Figure 3) and O (Figure 4) variants for 6 h, the levels of IL-6, IL-1β, and IL-18 were quantified using ELISA. The results demonstrated that stimulation with either variant significantly increased cytokine levels compared with the non-spike control group (*p* < 0.001). However, pretreatment with HPEE (25–100 μg/mL) and Luteolin (4.5–36 μM) for 24 h significantly diminished the release of these cytokines in a dose-dependent manner (*p* < 0.05).

Importantly, no statistically significant differences were detected between the Wuhan and Omicron variants in terms of their ability to induce inflammation or their response to HPEE and luteolin treatment. These findings indicate that HPEE and its active flavonoid, luteolin, effectively suppress spike protein-stimulated lung inflammation by inhibiting cytokine release, regardless of the viral variants.

### 3.4. Effects of HPEE and Luteolin on Inflammatory mRNA Expressions in SARS-CoV-2 Spike Proteins S1-Stimulated Lung Cell Inflammation

To further explore the anti-inflammation effects of HPEE and its active compound, luteolin, on spike protein-stimulated lung cell inflammation, we assessed their impact on inflammatory responses at the gene level by measuring the mRNA expression of key inflammation-related genes, including IL-6, IL-1β, IL-18, and NLRP3 in A549 lung epithelial cells.

**Figure 5:**
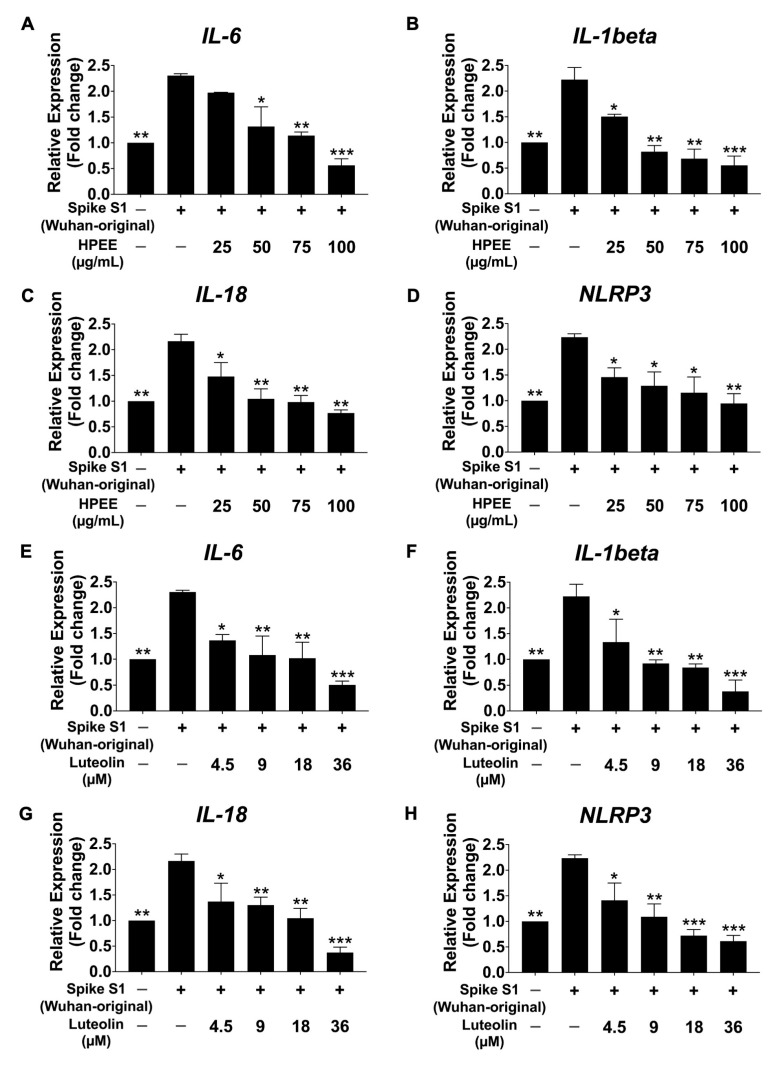
Effects of HPEE and luteolin on inflammatory mRNA expressions in Spike-S1 (Wuhan-original)-stimulated A549 cells. Cells were pretreated with HPEE (25–100 μg/mL: (**A**–**D**)) and luteolin (4.5–36 μM: (**E**–**H**)) for 24 h. The cells were then exposed to 100 ng/mL SARS-CoV-2 Spike S1 for 6 h. The mRNA expressions of *IL-6*, *IL-1β*, *IL-18*, and *NLRP3* were measured using RT-qPCR technique. The data (mean ± S.D.) was obtained from at least three independent experiments. * *p* < 0.05, ** *p* < 0.01, and *** *p* < 0.001 = statistical significance compared with the Spike S1-stimulated control group.

**Figure 6:**
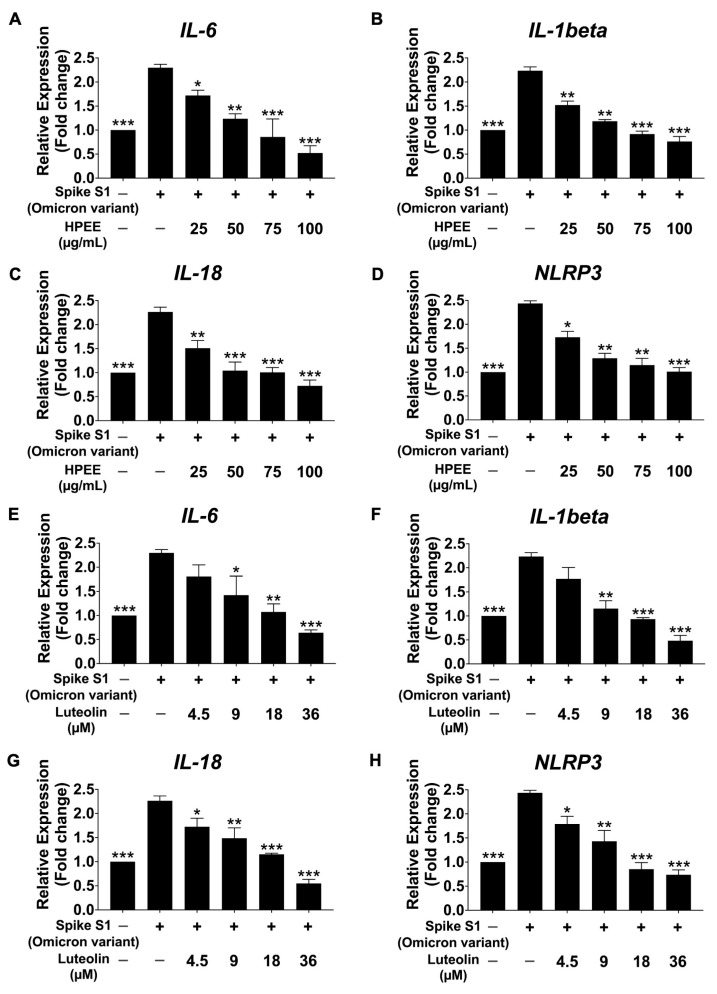
Effects of HPEE and luteolin on inflammatory mRNA expressions in Spike-S1 (Omicron variant)-stimulated A549 cells. Cells were pretreated with HPEE (25–100 μg/mL: (**A**–**D**)) and luteolin (4.5–36 μM: (**E**–**H**)) for 24 h. The cells were then exposed to 100 ng/mL SARS-CoV-2 Spike S1 for 6 h. The mRNA expressions of *IL-6*, *IL-1β*, *IL-18*, and *NLRP3* were measured using RT-qPCR. The data (mean ± S.D.) was obtained from at least three independent experiments. * *p* < 0.05, ** *p* < 0.01, and *** *p* < 0.001 = statistical significance compared with the Spike S1-stimulated control group.

Following 6 h of stimulation with spike protein S1 from both the Wuhan (Figure 5) and Omicron (Figure 6) variants, mRNA expression levels in SARS-CoV-2 spike protein-stimulated A549 cells were measured using qRT-PCR. The results revealed a significant upregulation of inflammatory mRNA expressions in both variants compared with the non-stimulated control group (*p* < 0.01), confirming the induction of an inflammatory response at the transcriptional level. Importantly, there were no statistical differences in the inflammatory response between the Wuhan and Omicron variants, suggesting that both variants induce similar inflammatory profiles in A 549 cells. However, pretreatment with varying concentrations of HPEE (25–100 μg/mL) and luteolin (4.5–36 μM) for 24 h significantly downregulated the inflammatory mRNA expression in a dose-dependent manner (*p* < 0.05). Therefore, HPEE and luteolin effectively attenuate spike protein-stimulated lung epithelial cell inflammation through the inhibition of inflammatory responses at the mRNA and protein levels, regardless of the variant.

### 3.5. Effects of HPEE and Luteolin on the NLRP3 Inflammasome Signaling in SARS-CoV-2 Spike Proteins S1-Stimulated Lung Cell Inflammation

The upregulates of inflammatory gene expression, promoting the synthesis and production of IL-1β and IL-18 outside the cells, was partly initiated by NLRP3 inflammasome signaling activation [34,35]. Given that both the Wuhan and Omicron variants stimulated similar inflammatory responses in A549 cells, we conducted mechanistic studies using the Wuhan variant to examine whether the anti-inflammation properties of HPEE and luteolin were regulated through the inhibition of spike protein-dependent-NLRP3 inflammasome activation by increasing the expression of inflammasome machinery proteins.

**Figure 7:**
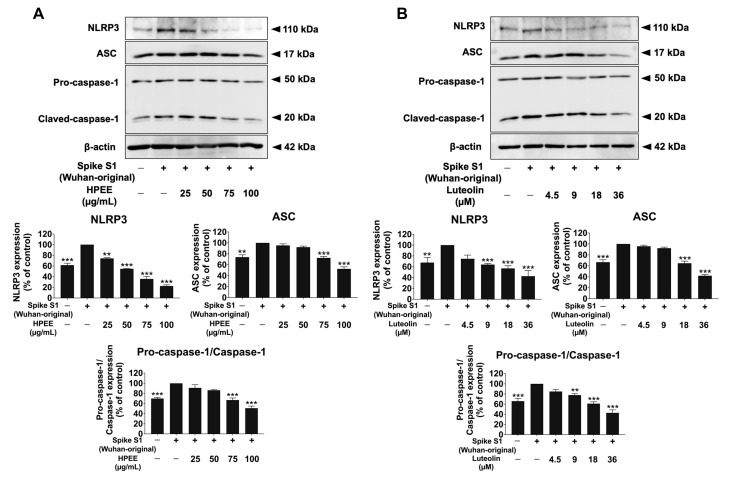
Attenuation of NLRP3 inflammasome signaling via downregulation of protein expressions in SARS-CoV-2 Spike-S1(Wuhan-original)-stimulated A549 lung cells by HPEE and luteolin. A549 cells were pretreated with HPEE (25–100 μg/mL: (**A**)) and luteolin (4.5–36 μM: (**B**)) for 24 h. Then, the cells were exposed to 100 ng/mL Spike S1 for 6 h. The data were visualized as band and the band density measurements using Western blot analysis. The band density measurement (mean ± S.D.) obtained from at least three independent experiments. ** *p* < 0.01, and *** *p* < 0.001 = statistical significance compared with the Spike S1-stimlated control group.

To assess this, we examined NLRP3 inflammasome-associated protein expressions in SARS-CoV-2 spike protein-stimulated A549 cells using Western blotting, as shown in Figure 7. The results demonstrated a significant upregulation of NLRP3, ASC, pro-caspase-1, and cleaved-caspase-1 in spike protein-stimulated A549 cells compared with the non-stimulated control group (*p* < 0.001, based on band density measurements). However, treatment with HPEE and luteolin significantly downregulated the expression of these proteins in a dose-dependent manner (*p* < 0.001). Overall, the anti-inflammation effects of HPEE and luteolin are mediated through inhibition of NLRP3 inflammasome activation.

### 3.6. Effects of HPEE and Luteolin on the MAPK Signaling in SARS-CoV-2 Spike Protein S1-Stimulated Lung Cell Inflammation

**Figure 8:**
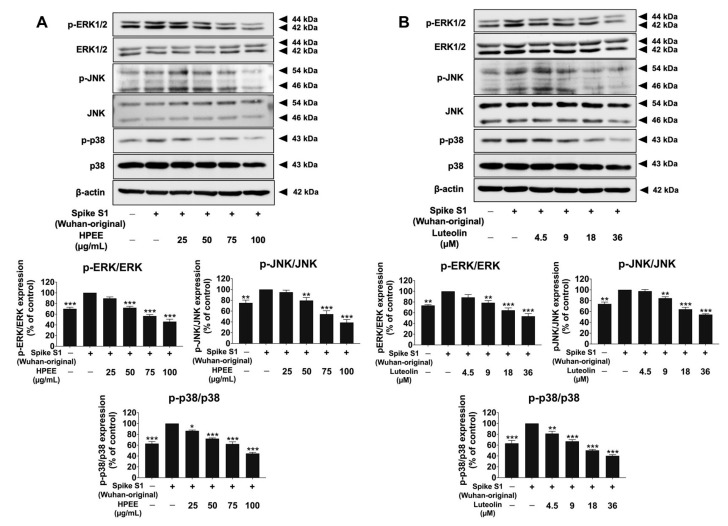
Attenuation of MAPK signaling via downregulation of protein expressions in SARS-CoV-2 Spike-S1(Wuhan-original)-stimulated A549 lung cells by HPEE and luteolin. A549 cells were pretreated with HPEE (25–100 μg/mL: (**A**)) and luteolin (4.5–36 μM: (**B**)) for 24 h. Then, the cells were exposed to 100 ng/mL Spike S1 for 6 h. The data were visualized as bands, and the band density measurements using Western blot analysis. The band density measurement (mean ± S.D.) obtained from at least three independent experiments. * *p* < 0.05, ** *p* < 0.01, and *** *p* < 0.001 = statistical significance compared with the Spike S1-stimlated control group.

To investigate the upstream regulatory mechanisms responsible for the anti-inflammation effects of HPEE and luteolin in spike protein-stimulated inflammation, we examined the expression of signaling proteins in the MAPK signaling pathway by employing Western blot analysis [36,37]. The resulting data is shown in Figure 8; the phosphorylation intensity determined by the band density levels of ERK, JNK, and p38 was significantly augmented in Spike S1-stimulated A549 cells compared to the non-stimulated control group (*p* < 0.001). However, pretreatment with HPEE and luteolin significantly reduced the phosphorylation of these MAPK-related proteins compared to the Spike S1-stimulated group (*p* < 0.001). Conclusively, these findings suggest that the anti-inflammation effects of HPEE and luteolin in SARS-CoV-2 Spike S1-stimulated lung cell inflammation are, in part, mediated through inhibition of the MAPK signaling pathway. The suppressions, along with the downregulation of the NLRP3 inflammasome triggering, likely contribute to the attenuation of inflammatory gene expressions and cytokine release.

## 4. Discussion

**Figure 9:**
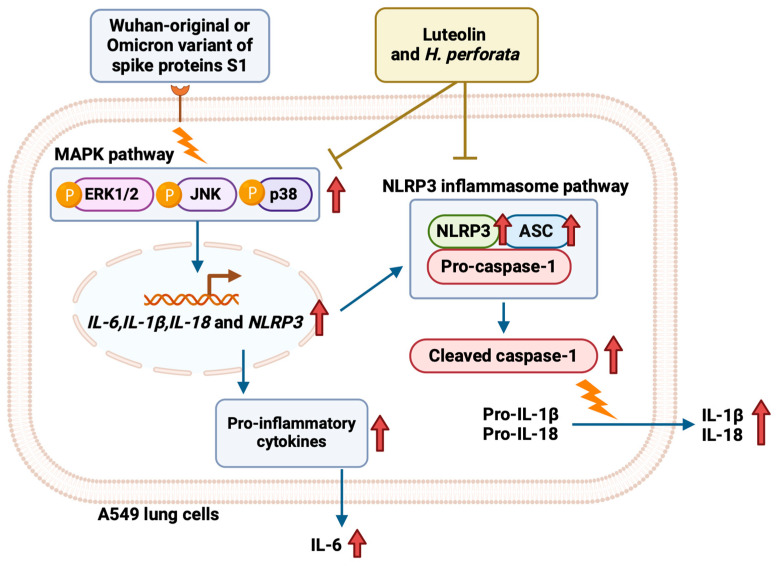
Schematic proposed mechanism of luteolin and HPEE attenuated SARS-CoV-2 Spike S1-stimulated A549 airway epithelial cells inflammation through inactivation of the ERK/JNK/p38 MAPK and NLRP3 inflammasome signaling pathway.

This study demonstrates that *H. perforata* ethanol extract (HPEE) and its major flavonoid, luteolin, exert potent anti-inflammation effects upon SARS-CoV-2 spike protein-stimulated lower airway inflammation using A549 lung epithelial cells. Specifically, our findings indicate that both HPEE and luteolin significantly hinder the release of inflammatory cytokines in A549 cells. Mechanistic analysis reveals that these effects are mediated through the inhibition of the MAPK/NLRP3 inflammasome signaling. The accomplish inhibitory mechanism of HPEE and Luteolin upon Spike S1-stimulated inflammation in A549 cells is displayed in Figure 9. Importantly, these inhibitory effects were observed across Wuhan and Omicron variants of SARS-CoV-2 spike proteins, signifying that HPEE and luteolin are broadly effective against spike protein-stimulated inflammation, irrespective of variant-specific mutations.

The spike protein part of SARS-CoV-2 is a well-known immune responses activator, particularly through toll-like receptors (TLRs), which generate downstream inflammatory signaling pathways [30,38]. One of the key mediators of spike protein-stimulated inflammatory reactions is the NLRP3 inflammasome, a cytosolic multiprotein complex that facilitates pro-casepase-1 being cleaved into its active caspase-1, led to IL-1β and IL-18 processing and cytokine releases. Excessive or chronic stimulation of NLRP3 inflammasome has been implicated in inflammation-associated COVID-19 pathology and long-term lung inflammation-associated PASC [39,40]. Our results show that both HPEE and luteolin effectively inhibit the core NLRP3 inflammasome machinery components, thereby reducing cytokine production. Long COVID-19 is associated with persistent inflammation contributing to ongoing symptoms following SARS-CoV-2 infection [41]. An increasing number of individuals report long-term symptoms, commonly including respiratory distress, muscle weakness, and neurological complications [42]. Several mechanisms have been proposed, notably involving cytokine dysregulation and activation of the NLRP3 inflammasome pathway [43,44]. Recent findings from the Blood Atlas Consortium of COVID-19 multi-omics highlight that immune signatures during long COVID-19 are often organ-specific, with evidence of inflammatory cell infiltration and inflammasome-related gene networks involving ribosomal proteins and key genes associated with inflammasome function, such as *NLRP*, *MAP3K14*, and *FOXP1* [45]. These findings are supported by preceding studies demonstrating the anti-inflammation effects of flavonoids, such as luteolin, in mitigating inflammasome activation in other inflammation conditions [20,46,47,48].

Flavonoids, the second metabolites derived from plants, are commonly recognized for the various beneficial properties, including anti-oxidative stress, anti-inflammation, anti-diabetic, anti-allergic, and anti-cancerous properties [29,49]. Recently, molecular docking studies revealed that numerous flavonoids from plants, such as catechin, epicatechin, epigallocatechin gallate, kaempferol, quercetin, curcumin, anthocyanins, and luteolin, were effective anti-SARS-CoV-2 agents by [50,51]. In our study, identification of the major flavonoid compounds in HPEE was performed using HPLC with a technique with eight standard flavonoids (catechin, epicatechin, hyperoside, rutin, quercitrin, quercetin, luteolin, and apigenin) [27,52]. Among these, luteolin was found to be the predominant compound in HPEE and exhibited potent anti-inflammatory effects and may offer therapeutic potential against spike protein-induced lung inflammation. However, this does not represent the complete phytochemical profile of the extract. Previous studies have reported other bioactive compounds in *H. perforata* roots, including limonoids and chromones with notable anti-inflammatory effects [53]. Briefly, haperforatone F limonoid and harriforatin chromone were recognized as nitric oxide production inhibitors in LPS-induced murine macrophages [54,55]. Thus, comprehensive profiling of HPEE using high-throughput identification techniques with spectrophotometry such as LC-MS and UPLC-MS/MS is required. These techniques could help confirm the component found in HPEE and eliminate other compounds with the same hydrophobicity that could influent the identification using retention time of chromatogram from HPLC technique, and it could potentially reveal other biologically active compounds, such as limonoids, chromone, and alkaloids, that are worthy of further investigation for anti-inflammation agents of SARS-CoV-2 spike protein-induction. Moreover, these additional components could be involved in an overall anti-inflammatory activity observed in SARS-CoV-2 spike protein-stimulated lung inflammation and merit further investigation. Our previous study identified luteolin as a major compound in the ethyl acetate fraction of *Perilla frutescens*, with a concentration of 248.82 ± 12.34 mg/g extract [20]. However, our current study, the crude extract obtained through ethanolic extraction of *H. perforata* was found to contained a high amount of luteolin, measuring 143.53 ± 1.58 mg/g extract. The high luteolin content in ethanolic extracts provides a practical approach for further applications due to its ease of extraction, biocompatibility, and safety [56,57]. Moreover, previous studies on the stability of phytochemical compounds in ethanolic extract reveal that the ethanolic extract can withstand the change of thermal and humidity as well as the stability of bioactive compounds is maintained when stored at room temperature for at least 2 years [58,59]. Nevertheless, confirmation of our extract regarding the stability should also be conducted in order to confirm the real-world application of the extract in in vivo and clinical studies.

To examine the anti-inflammation properties of the active flavonoid compounds from *H. perforata*, the concentration range of luteolin was calculated based on HPLC analysis. Therefore, in our experimentations, 0–140 μM concentrations of luteolin were representative of the quantity identified in HPEE (0–200 μg/mL). Additionally, we determined the cytotoxicity of *H. perforata* ethanolic extract and luteolin on A549 and primary dermal fibroblast cells using the SRB assay. The IC_50_ value for HPEE on A549 cells in our study (IC_50_ > 200 μg/mL) was comparable to those reported in existing literature, where IC_50_ value > 50 μg/mL or IC_50_ value > 100 μg/mL were observed for ethanolic extracts of *H. perforata* [23,60]. Similarly, the IC_50_ values of luteolin on A549 cells reported in other studies ranged from >70 μM to >200 μM [20,61]. These previously reported IC_50_ values are consistent with our findings (IC_50_ = 101 ± 7.57 μM). Based on these prior data and our current results, non-toxic concentrations of HPEE (25–100 μg/mL) and luteolin (4.5–36 μM) were used to investigate their anti-inflammation properties against the spike protein-stimulated NLRP3 inflammasome activation in A549 cells.

Our study found that HPEE and luteolin function as inflammatory inhibitors upon SARS-CoV-2 spike protein-stimulated A549 cells inflammation by suppressing inflammatory cytokine release. Consistent with our findings, luteolin has been widely reported for its anti-inflammatory effects. For instance, luteolin and *P. frutescens* seed extracts, as investigated in our previous study, inhibited Spike S1-induced inflammation (Wuhan-original variants) by downregulating JAK/STAT-mediated signaling and suppressing the release of inflammatory cytokines in lung cells [20]. Luteolin at 3–10 μM suppressed the IL-6, IL-1β, and IL-8 inflammatory genes in THP-1 macrophages [62,63]. Additionally, luteolin inhibited spike glycoprotein S1-stimulated inflammation in macrophage-derived cell lines through the ER stress-induced calcium-CHOP-MAPK signaling [48]. In the current study, we expanded our investigation to examine the broader anti-inflammatory potential of luteolin and *H. perforata* extracts against multiple Spike S1 variants, including both the original Wuhan and Omicron variants in A549 lung cells, with a focus on the inhibition of ERK, JNK, and p38 MAPK signaling pathways. Overall, these findings indicate that luteolin—regardless of its plant source—exhibits broad-spectrum anti-inflammatory effects through the modulation of multiple pathways, including JAK/STAT and MAPK. These insights provide valuable information for future in vivo investigations and clinical applications.

Regarding the clinical relevance of the doses used in our study for both HPEE and luteolin that successfully inhibited the SARS-CoV-2 Spike S1-stimulated A549 airway epithelial cells inflammation, the concentration of HPEE (25–100 μg/mL) was based on the previous studies investigating the anti-inflammation properties of the ethanolic root extract of *H. perforata* in LPS-induced macrophage cell experiments and carrageenan-induced inflammation in Wistar rats. It was reported that *H. perforata* extract at concentrations of 3.125–50 μg/mL inhibited IL-1β and IL-6 mRNA expression. These concentrations were subsequently translated and applied in vivo in a similar study, corresponding to doses of 50–400 mg/kg, which demonstrated anti-inflammatory effects [25]. For luteolin, a dose range up to 36 μM has been validated for its protective effects in the acute lung injury model of mice. Doses of 18, 35, and 70 μmol/kg (equivalent to approximately 18, 35, and 70 μM in vitro) were shown to alleviate LPS-induced lung inflammation through suppression of IL-6 and TNF-α in bronchoalveolar lavage fluid samples [64,65]. Therefore, both HPEE and luteolin exhibit promising anti-inflammatory activity at concentrations that are translatable between in vitro and in vivo models, supporting the conceivable candidates for further development and evaluation in clinical studies targeting chronic inflammatory diseases.

Furthermore, we found that HPEE and luteolin suppress the MAPK signaling pathway, particularly by reducing ERK, JNK, and p38 MAPK protein phosphorylations. The MAPK cascade is a critical regulator of inflammatory response and is commonly known to be activated by viral infections, including SARS-CoV-2 [18,66]. Many reports have shown that spike protein-mediated MAPK pathway signaling activation contributes to the sustained pro-inflammatory cytokine release, thereby exacerbating lung inflammation [67,68]. By inhibiting MAPK signaling, HPEE and luteolin likely disrupt the amplification of inflammatory signals, further supporting their potential as therapeutic agents. Consistent with our findings, studies have shown that hindering p38 with the SB203580 MAPK inhibitor could suppress airway respiratory tract inflammation in vivo and reduce NLRP3 inflammasome expression and IL-1β release in an acute lung injury-stimulated mouse model [69]. Luteolin has been described as a small naturally occurring molecule that downregulates the ER stress-MAPK pathway by modulating the phosphorylation of MAPK-related proteins in spike protein-stimulated inflammation scenarios in THP-1 immune macrophage cells [48]. Additionally, luteolin was found to inhibit MAPK and NF-κB pathways and attenuate the pulmonary inflammatory response in mouse models [69]. Given that our proposed inhibitory mechanism of HPEE and luteolin on SARS-CoV-2 spike protein-stimulated inflammation involves modulation of TLRs and MAPK/NLRP3 inflammasome pathways, further upstream validation is warranted. This includes using TLR knockdown models or selective pharmacological inhibitors, along with NF-κB reporter assays, to confirm the specificity and potential crosstalk between these signaling pathways in SARS-CoV-2-spike protein-induced inflammation.

The broad-spectrum anti-inflammation effects observed against both Wuhan and Omicron spike proteins highlight the potential of HPEE and luteolin as candidate treatments for long COVID-19-associated lung inflammation. Despite the substantial mutations in the Omicron variant that enhance immune evasion and alter viral pathogenesis [70], our findings indicate that the fundamental inflammatory response remains largely conserved. This suggests that targeting common downstream inflammatory pathways, such as the NLRP3 inflammasome and MAPK signaling, could be a viable strategy for mitigating spike protein-stimulated inflammation across different SARS-CoV-2 variants.

The drawback of this study is that the experiments were conducted in vitro using A549 lung epithelial cells. While these cells provide a relevant model for studying lung inflammation [30,71], many studies utilized the airway epithelial cells expressing the ACE2 receptor, including human bronchial epithelial cells (HBEpC), type II pneumocyte lung epithelial cells (A549 cells), or the lung cancer cell line (Calu-3 cells) for investigating of spike glycoprotein-induced inflammation. While the cells provide a relevant model for studying lung inflammation, further validation in immune cells, animal models, or clinical studies is necessary to confirm the in vivo efficacy of HPEE and luteolin. Additionally, future experiments using other immune-relevant cell lines, including THP-1 macrophages and primary bronchial epithelial cells, would strengthen the translational relevance of our findings. Additionally, future studies should investigate whether these compounds influence other inflammatory pathways involved in long COVID-19, including NF-kB and oxidative stress signaling pathways.

## 5. Conclusions

Overall, this study demonstrates that *Harrisonia perforata* ethanolic extract (HPEE) and its major flavonoid, luteolin, effectively suppress SARS-CoV-2 Spike S1 protein-stimulated A549 lung cells inflammation by inhibiting the MAPK/NLRP3 inflammasome signaling pathways. Both HPEE and luteolin reduced pro-inflammatory IL-6, IL-1β, and IL-18 cytokine genes and proteins with consistent efficacy across SARS-CoV-2 spike proteins from both the Wuhan and Omicron variants, with minimal cytotoxicity. The high luteolin content in HPEE supports its therapeutic potential, and our findings could encourage the application of natural compounds as candidate interventions for long COVID-19-associated lung inflammation. Further in vivo and in-depth mechanistic studies are warranted to advance their use in clinical settings.

## Figures and Tables

**Table 1:** Gradient condition for HPEE’s flavonoid compound analysis using HPLC.

Retention Time (RT: min)	Mobile Phase A	Mobile Phase B
0 (Start)	70%	30%
0.01–5	70–65%	30–35%
5.01–40	65–60%	35–40%
40.01–50	60–55%	40–45%
50.01–75	55–54%	45–46%
75.01–80	54–40%	46–60%
80.01–85	40%	60%
85.01–90	40–70%	60–30%

**Table 2:** HPEE Phytochemical characteristics and flavonoid constitutes.

Phytochemicals	mg/g of Extract Unit (Mean ± S.D.)
Total flavonoid (mg of catechin equivalents/g extract)	152.17 ± 14.56
Apigenin	2.34 ± 0.22
Catechin	ND
Epicatechin	8.22 ± 0.57
Hyperoside	3.33 ± 0.70
Luteolin	143.53 ± 1.58
Quercetin	ND
Quercitrin	ND
Rutin	ND

ND = not detectable. Data are displayed as mean ± S.D. values of at least three replicates.

**Table 3:** The IC_50_ values of HPEE and luteolin in A549 cells.

A549 Cells	HPEE (µg/mL)	Luteolin (µM)
24 h	>200	101 ± 7.57
48 h	178 ± 26.87	34 ± 1.53

## Data Availability

Data are contained within this article and Supplementary Materials.

## Supplementary Materials

The following supporting information can be downloaded at: https://www.mdpi.com/article/10.3390/life15071077/s1; Figure S1: Effects of Spike S1-stimulated inflammatory cytokine release in A549 cells; Figure S2: Effects of Spike S1-stimulated inflammatory cytokine mRNA expressions in A549 cells; Table S1: Primer sequences were used in this study for the determination of gene expressions by RT-qPCR analysis.
