# Supplementary material for: Luteolin-Rich Extract from Harrisonia perforata (Blanco) Merr. Root Alleviates SARS-CoV-2 Spike Protein-Stimulated Lung Inflammation via Inhibition of MAPK/NLRP3 Inflammasome Signaling Pathways
Source: Life (Basel). 2025 Jul 5;15(7):1077. doi: 10.3390/life15071077 (PMC12299524; doi:10.3390/life15071077)
Supplement: Supplementary file 1 [file life-15-01077-s001.zip › life-3715023-supplementary.pdf]

Supplementary data

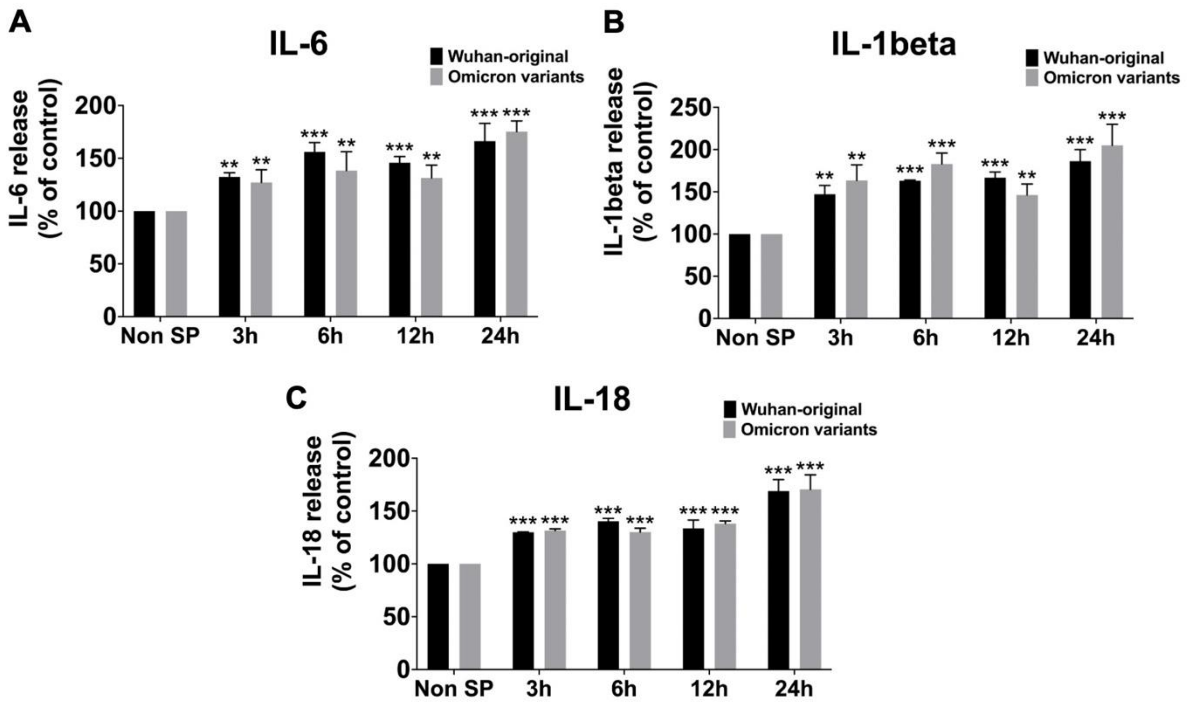

Figure S1. Effects of Spike S1-induced inflammatory cytokine release in A549 cells. A549 cells were exposed to 100 ng/mL of Spike S1, Wuhan original and Omicron variants for 0-24 h. The levels of IL-6 (A), IL-1β (B), and IL-18 (C) released into the culture supernatant were measured using ELISA. The presented data represent the mean ± S.D. values obtained from at least three independent experiments performed in triplicate. ** $p < 0.01$, and *** $p < 0.001$ indicate statistically significant differences compared with the Spike S1-induced control group.

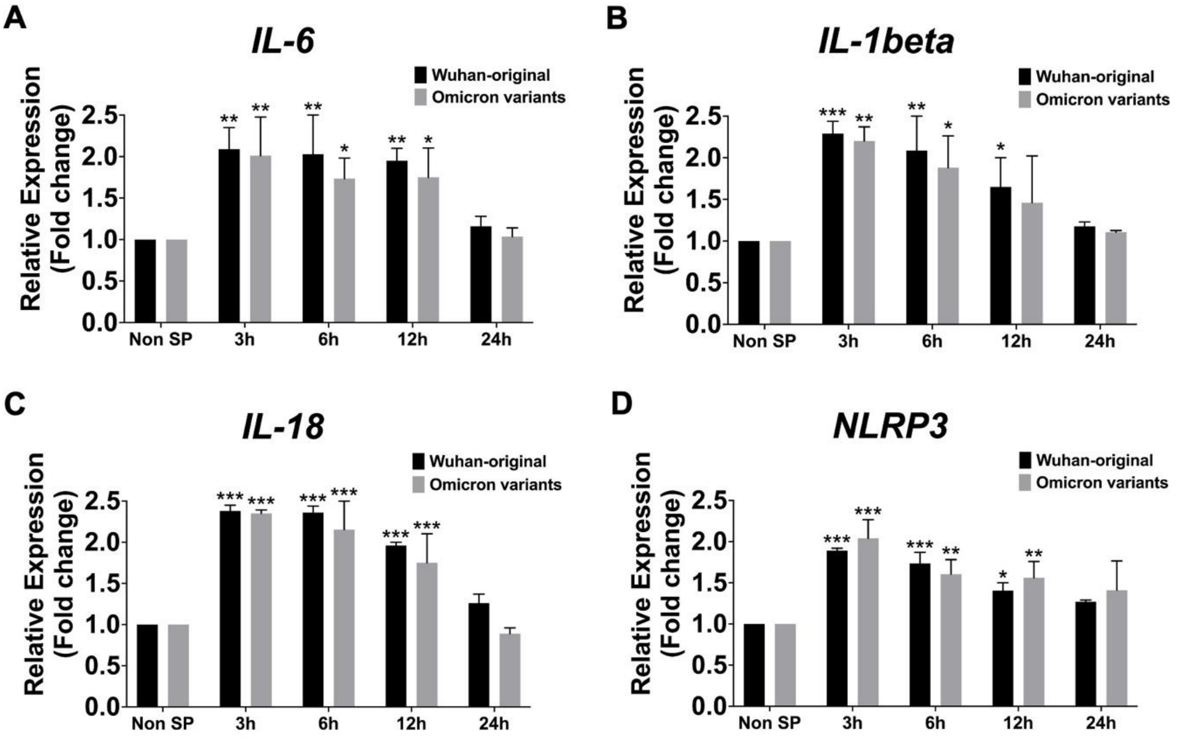

Figure S2. Effects of Spike S1-induced inflammatory cytokine mRNA expressions in A549 cells. A549 cells were exposed to 100 ng/mL of Spike S1, Wuhan original and Omicron variants for 0-24 h. The mRNA expressions of *IL-6* (A), *IL-1β* (B), *IL-18* (C), and *NLRP3* (D) were assessed using RT-qPCR. The presented data represent the mean ± S.D. values obtained from at least triplicate independent experiments; * $p < 0.05$, ** $p < 0.01$, and *** $p < 0.001$ indicate statistically significant differences compared to the Spike S1-induced A549 cells.

Table S1. Primer sequences were used in this study for the determination of gene expressions by RT-qPCR analysis.

Gene product	Primer sequences
IL-6	Forward: 5′-ATG AAC TCC TTC ACA AGC-3′<br>Reverse: 5′-GTT TTC TGC CAG TGC CTC TTT G-3′
IL-1β	Forward, 5′- TGC TCA AGT GTC TGA AGC AG-3′<br>Reverse, 5′- TGG TGG TCG GAG ATT CGT AG -3′
IL-18	Forward, 5′- TCG GGA AGA GGA AAG GAA CC-3′<br>Reverse, 5′- TTC TAC TGG TTC AGC AGC CA - 3′
NLRP3	Forward, 5′-AAC ATG CCC AAG GAG GAA GA -3′<br>Reverse, 5′- GGC TGT TCA CCA ATC CAT GA -3′
GAPDH	Forward, 5′- TCA ACA GCG ACA CCC AC -3′<br>Reverse, 5′- GGG TCT CTC TCT TCC TCT TGT G-3′
